# Poor Control of Plasma Triglycerides Is Associated with Early Decline of Estimated Glomerular Filtration Rates in New-Onset Type 2 Diabetes in China: Results from a 3-Year Follow-Up Study

**DOI:** 10.1155/2020/3613041

**Published:** 2020-09-28

**Authors:** Chuan Wang, Lingshu Wang, Kai Liang, Fei Yan, Xinguo Hou, Fuqiang Liu, Li Chen

**Affiliations:** ^1^Department of Endocrinology, Qilu Hospital of Shandong University, Jinan, Shandong, 250012, China; ^2^Institute of Endocrine and Metabolic Diseases of Shandong University, Jinan, Shandong, 250012, China; ^3^Key Laboratory of Endocrine and Metabolic Diseases, Shandong Province Medicine & Health, Jinan, Shandong, 250012, China; ^4^Jinan Clinical Research Center for Endocrine and Metabolic Diseases, Shandong Province Medicine & Health, Jinan, Shandong, 250012, China

## Abstract

**Objective:**

Diabetic kidney disease (DKD) is the most common cause of end-stage renal disease (ESRD). Even after strict control of obesity, hyperglycemia, and hypertension, some patients still progress rapidly. Previous studies suggested diabetic dyslipidemia might be one of the factors responsible for this high residual risk. This study aims to explore the impact of long-term lipid control on renal outcome in new-onset type 2 diabetes mellitus (T2DM).

**Methods:**

We conducted a 3-year follow-up study, involving 283 subjects with new-onset T2DM, and observed the effect of baseline and follow-up metabolic abnormalities, especially dyslipidemia, on the early damage of kidney function using multiple logistic regression analysis.

**Results:**

After 3 years follow-up, patients achieved a better control of body weight, hypertension, and blood glucose. The most reduced eGFR group shared the least reduced BMI and LDL-C, as well as the greatest increase in TG levels. Only TG in the follow-up, not any of the baseline data, nor obesity, blood glucose, BP, or LDL-C in the follow-up, was found to be significantly correlated with the most reduced eGFR. Compared with patients with constantly abnormal TG levels, the risks were even higher in the subjects who experienced a transition from normal TG to hypertriglyceridemia (OR = 2.576 versus OR = 2.184, after multiple adjustment), and by tight controlling of TG, patients started with abnormal baseline TG levels could reduce the risk of DKD progression to the same low levels as the TG-constantly-normal group.

**Conclusion:**

This study emphasized the importance of long-term TG control in East Asian patients with new-onset T2DM: TG control can delay the decline of kidney function in the early stage of DKD, and reversal of hypertriglyceridemia may undo the risks of the past. It is time to pay more attention to the control of TG in new-onset T2DM.

## 1. Introduction

Diabetic kidney disease (DKD) is one major microvascular complication of type 2 diabetes mellitus (T2DM) and has been the most common cause of end-stage renal disease (ESRD) in Western countries [1], which ranks the second in China [2]. From 2009 to 2012, the prevalence of DKD in China was 30%-50% in community diabetic patients and about 40% in hospitalized patients [3], while in 2017 to 2018, the overall prevalence of CKD in patients with diabetes was 48.0% [4]. The progression of renal function decline in DKD is insidious and rapid, and lacking effective treatment for ESRD makes it very important to find and control risk factors for DKD at a stage as early as possible [5].

Numerous studies have proved the important roles of obesity, hyperglycemia, and hypertension in the development and progression of DKD [6–8]. However, even under strict control of the above risk factors, some patients still progress to ESRD, and diabetic dyslipidemia (high triglycerides (TG) and/or low high-density lipoprotein cholesterol (HDL-C)) may be one of the factors responsible for this high residual risk [9]. Actually, the associations of TG and HDL-C with the development of DKD had been investigated by several studies [10, 11]. However, these studies mainly focused on the relationship between baseline lipid levels and DKD in subjects with previously diagnosed T2DM; few studies explored the impact of enhanced lipid control on the renal outcome in new-onset diabetes. In particular, evidence of whether long-term TG control improves renal outcome, especially in new-onset diabetes, is still lacking. In order to clarify this problem and achieve the early identification, warning, and control of DKD, we conducted a 3-year follow-up study to observe the effect of baseline and follow-up metabolic abnormalities, especially dyslipidemia, on the early damage of kidney function in new-onset T2DM patients.

## 2. Methods

### 2.1. Participants

This study was one part of the Risk Evaluation of Cancers in Chinese Diabetic Individuals: a Longitudinal (REACTION) study [12]. In the 2012 baseline survey, we recruited 10,028 subjects in Shandong Province, China. A 3-year follow-up was performed at 2015, which included 4778 subjects who participated in the on-site, 2864 subjects who completed a telephone survey, 159 subjects who died, and 2227 subjects who were lost to follow-up, which yielded a follow-up rate of 77.8%. Of the 4778 subjects who participated in the on-site follow-up, 523 new-onset T2DM patients were diagnosed based on ADA diagnostic criteria [13]. Then, subjects with the following traits were excluded: (1) missing data for calculating estimated glomerular filtration rate (eGFR), (2) eGFR <60 mL/min/1.73 m^2^, (3) △eGFR ≥0 mL/min/1.73 m^2^, (4) previously diagnosed hypertension or dyslipidemia, and (5) history of any kidney disease, hepatic disease, or malignant disease. Ultimately, 283 subjects (including 195 women) were eligible for the analysis. The study protocol was approved by the institutional review board at the Department of Endocrinology and Metabolic Disease, Ruijin Hospital, Shanghai Jiaotong University School of Medicine. All subjects provided informed consent.

### 2.2. Data Collection and Measurements

The methods of data collection and measurements have been described in our previous study [14, 15]. In general, body mass index (BMI) was calculated as weight (kg) divided by height squared (m^2^). Fasting venous blood samples were collected for measurement of fasting blood glucose (FBG), HbA1c, TG, HDL-C, low-density lipoprotein cholesterol (LDL-C), and creatinine by use of an automatic analyzer. Postprandial blood glucose (PBG) was measured after subjects had completed a 75 g oral glucose tolerance test (OGTT). The eGFR was calculated by Chronic Kidney Disease Epidemiology Collaboration (CKD-EPI) equation [16]. △eGFR = follow − up eGFR minus baseline eGFR.

### 2.3. Definition

Based on ADA diagnostic criteria [13], overweight/obesity was defined by BMI ≥25 kg/m^2^; hypertension was defined by systolic BP/diastolic BP ≥140/90 mmHg; diabetes was defined by FBG ≥7.0 mmol/L and/or PBG ≥11.1 mmol/L and/or HbA1c ≥6.5%, and poor glycemic control was defined by HbA1c ≥6.5%; high TG was defined by TG ≥1.70 mmol/L; low HDL-C was defined by <1.0 mmol/L for men or <1.3 mmol/L for women; out of target for LDL-C was defined by LDL-C ≥2.6 mmol/L. Besides, all subjects were divided into three groups based on the tertile value of △eGFR, and the highest △eGFR tertile was defined as the most reduced eGFR.

### 2.4. Statistical Analysis

The continuous variables with normal distribution in baseline and follow-up survey were presented as the means ± standard deviation (SD). However, the changes of metabolic indicators and eGFR were skewed distribution, so these variables were expressed as the median (interquartile range). The categorical variables were presented as numbers (%). Differences between groups were analyzed using Student's *t*-test for continuous data with normal distribution, Kruskal-Wallis H test for skewed continuous variables, and the chi-square test for categorical data. Spearman correlation analysis was used to explore the relationships between △ (metabolic indicators) and △eGFR. The associations of baseline and follow-up metabolic abnormalities with the most reduced eGFR were estimated using multiple logistic regression analysis in different models. Finally, we divided all subjects into four groups based on the TG value at baseline and follow-up: G1 (baseline: normal TG; follow-up: normal TG), G2 (baseline: high TG; follow-up: normal TG), G3 (baseline: normal TG; follow-up: high TG), and G4 (baseline: high TG; follow-up: high TG). Then, the associations of different TG group with the risk of most reduced eGFR were estimated using multiple logistic regression analysis in different models. *P* values < 0.05 were considered statistically significant. All statistical analyses were performed using SPSS 22.0 (SPSS Inc., Chicago, IL, USA).

## 3. Results

### 3.1. Comparison of the Baseline and Follow-Up Characteristics in New-Onset T2DM Subjects

The demographics and lab results of study participants were shown in Table 1. The results of the follow-up showed a better control of body weight, hypertension, and blood glucose with decreased BMI, BP, and HbA1c. The above results might be due to the enhanced management of patients during the follow-up, including the usage of hypoglycemic agents and antihypertensive agents; although, we did not collect the medication history during follow-up. Besides, we observed that both of HDL-C and LDL-C decreased during follow-up, while TG did not exhibit significant change.

### 3.2. Baseline and Follow-Up Characteristics in Study Participants Divided by △eGFR

To explore the possible related factors of renal function decline in new-onset T2DM, we first divided the study subjects into three groups based on the tertile value of △eGFR and compared the clinical characteristics between groups, as shown in Table 2. The highest △eGFR tertile was defined as the most reduced eGFR. It appeared that the most reduced eGFR group shared the least reduced BMI and LDL-C, but there was no significant difference in blood glucose and BP control between groups. More importantly, the statistics also showed the most reduced eGFR group exhibited the greatest increase in TG levels. These results suggested that the poor control of BMI, LDL-C, and TG might be related to the progression of DKD in new-onset T2DM.

### 3.3. Spearman Correlation Analysis of the Risk Factors of △eGFR

In order to investigate the correlation between the eGFR decline and the change of metabolic indicators, we conducted a Spearman correlation analysis, as shown in Table 3. It appeared that △BMI (*r* = −0.204, *P* = 0.001), △TG (*r* = −0.155, *P* = 0.009), △LDL-C (*r* = −0.165, *P* = 0.005), and △FBG (*r* = −0.178, *P* = 0.003) were all negatively correlated with △eGFR, suggesting that the more reduction of body weight, blood lipid, and blood glucose; the more benefit we can get to delay the progress of DKD.

### 3.4. The Associations of Baseline and Follow-Up Metabolic Abnormalities with the Most Reduced eGFR

Next question is which metabolic abnormality is more relevant to the most decline of eGFR, the baseline data or the follow-up? To address this issue, we estimated the associations of baseline and follow-up metabolic abnormalities with the most reduced eGFR using the multiple logistic regression analysis. The results were shown in Table 4. Surprisingly, no significant correlation was found in any of the baseline data with the most reduced eGFR, regardless of how the independent variables were analyzed, separately (Model 1) or simultaneously (Model 2). Meanwhile, TG in the follow-up, not obesity, blood glucose, BP, or LDL-C, was found to be significantly correlated with the most reduced eGFR. This result emphasized the importance of long-term TG control: compared to the baseline, the follow-up TG levels might be of more importance for the disease course of DKD.

### 3.5. Multiple Logistic Regression Analysis of Different TG Control Status with the most Reduced eGFR

Finally, we want to explore whether subjects who have had hypertriglyceridemia at baseline and turned to be normal during the follow-up share the same risk with people whose TG levels are constantly normal. We divided all subjects into four groups based on the TG value at baseline and follow-up: G1 (baseline: normal TG; follow-up: normal TG), G2 (baseline: high TG; follow-up: normal TG), G3 (baseline: normal TG; follow-up: high TG), and G4 (baseline: high TG; follow-up: high TG). Then, the associations of the different TG group with the risk of most reduced eGFR were estimated using multiple logistic regression analysis after adjusting for age, gender, overweight/obesity, hypertension, poor glycemic control, high TG, low HDL-C, and out of target for LDL-C, as shown in Table 5. The results indicated that as long as hypertriglyceridemia existed in the follow-up, there was a risk of rapid DKD progression; compared with patients with constantly abnormal TG levels, the risks were even higher in the subjects who experienced a transition from normal TG to hypertriglyceridemia (OR = 2.576 versus OR = 2.184, in Model 3). The above results emphasized the urgency of TG control: the elevated TG level in the follow-up was an independent risk factor for the rapid development of DKD. On the bright side, it also brought hope: even patients started with abnormal baseline TG levels could reduce the risk of DKD progression to the same low levels as the TG-constantly-normal group, by tight controlling of TG. It is not too late to start to control TG for new-onset T2DM, and the benefit is rewarding.

## 4. Discussion

DKD has been the most common cause of ESRD in Western countries, and numerous risk factors have been identified for its progression, including but not limited to older age, smoking, alcohol consumption, overweight/obesity, hyperglycemia, hypertension, dyslipidemia, and hyperuricemia [4, 17, 18]. However, even under strict control of body weight, blood glucose, and blood pressure, some patients still progress to ESRD. Therefore, it is very important to find and manage risk factors for DKD as precise as possible.

Previous studies had shown that dyslipidemia was a well-established risk factor for the renal impairment in diabetes; it is not only associated with the occurrence [2, 10, 19] but also the deterioration [4, 11] of DKD. A cross-sectional study published in 2014 involving 13 countries reported the plasma lipid's contribution to the occurrence of DKD; hypertriglyceridemia significantly increased the risk of DKD, and high HDL-C was a protective factor [10]. Another multicentered, large-scale study with a 4 years follow-up also suggested that TG ≥150 mg/dL increased the risk of an eGFR reduction >30% by 29%, HDL-C <40 mg/dL in men and <50 mg/dL in women were associated with a 28% increased risk of eGFR reduction [11]. Our group also reported baseline TG levels were closely associated with a mildly reduced eGFR in community subjects with normal serum lipid levels [15]. Of course, there was also an article reported that plasma lipid was not associated with eGFR decline in T2DM after multivariable adjustment [20]. However, all the above studies focused on the baseline lipid levels and were conducted in the subjects with previously diagnosed T2DM, relatively few studies would discuss the impact of enhanced lipid control on the renal endpoints [21], and even fewer based their studies on the influence of lipid control on renal dysfunction in new-onset T2DM.

Evidences about lipid control improving DKD renal endpoints were mainly regarding the management of cholesterol: keeping HDL-C greater than 50 mg/dL for women and greater than 40 mg/dL for men could prevent the development of new-onset microalbuminuria (HR = 0.715) [21]. ABCD-RA Clinical Practice Guidelines (2017) did suggest to apply lipid-lowering therapy in DKD with hypercholesterolemia; however, the major goal of which was to reduce risk of cardiovascular events rather than prevent ESRD. Some studies even found that achieving LDL-C goals through statin therapy was insufficient to meaningfully reduce the risk for developing renal dysfunction and the presence of elevated or high TG conferred an added risk of developing kidney disease in a large proportion of statin-treated patients with diabetes and/or hypertension [22, 23]. Until now, evidence of whether long-term TG control improves renal outcome, especially in new-onset T2DM patients, is still lacking. In this study, we conducted a 3-year follow-up and analyzed 283 new-onset T2DM patients. The results showed that hypertriglyceridemia in the follow-up was closely related to the high risk of rapid eGFR decline after multiple adjustments. Compared with patients with constantly abnormal TG levels, the risks were even higher in the subjects who experienced a transition from normal TG to hypertriglyceridemia, and patients started with abnormal baseline TG levels could reduce the risk of DKD progression to the same low levels as the TG-constantly-normal group, by tight controlling of TG.

TG's adverse impact to renal function has been proven by many basic studies. Ectopic deposition of TG-rich lipid droplets had been observed in renal biopsies of T2DM patients, practically the same as in animal models of T2DM [24, 25]. Accumulation of TG could activate monocytes, stimulate the secretion of TGF-*β*, and induce the production of reactive oxygen species et al., and these above cause the degradation of glycocalyx, increase of glomerular filtration barrier permeability, tubular injury, and interstitial fibrosis, thus resulting in DKD progression [24]. Lipotoxicity of TG could also cause mitochondrial dysfunction in the podocytes and in tubules, the subsequent ATP depletion, and fatty acid *β*-oxidation failure in turn lead to more lipid disposition, so did the vicious cycle begin [26]. Breaking the wretched cycle might have some effect in relieving kidney damage and preserving renal function. Intervention measures, such as liraglutide [27], disulfide-bond A oxidoreductase-like protein (DsbA-L) [28], berberine [29], and resveratrol [30], whose renal protection effects had been proved to be partly relied on reducing TG contents in the kidney. Here, in our study, we verified the above results and further emphasized the risk of TG for DKD progression and the necessity of controlling TG.

The significance of our study is to emphasize the importance of long-term TG control in East Asian patients with new-onset T2DM; TG control is not only important in previously diagnosed DM but also works in new-onset T2DM and can delay the decline of kidney function in the early stage of DKD. It also brings hope; reversal of hypertriglyceridemia can undo the risks of the past. TG is a handy intervention target. To most patients with mildly elevated TG levels, except for those who had familial hypertriglyceridemia, increasing dietary fiber and reducing fat intake can both effectively reduce TG. Therefore, TG intervention can be regarded as an effective and inexpensive target to prevent the progression of DKD to ESRD. Next, our research group plans to observe the effect of TG intervention to the renal endpoint by introducing a standard dietary protocol, and we want to explore the underlying mechanisms as well.

Of course, our study also had some limitations. Firstly, we only included Chinese in our study; the results might not apply to people of other ethnicities. Secondly, some factors that might affect the accuracy of the results were not adjusted in the models, including the medication history of hypoglycemic drugs, antihypertensive drugs, and lipid-lowering drugs. However, as a real-world study, the results actually reflected the current status of metabolic control in new-onset T2DM in China, no matter what treatment the patient took. Finally, some selection bias should also be considered. We only included patients with △eGFR ≤0 mL/min/1.73m^2^ in our study. However, since glomerular hyperfiltration was also the early sign of DKD, we could not determine whether his kidney function was impaired or not for patients with △eGFR >0 mL/min/1.73m^2^. Therefore, we excluded the patients who have △eGFR >0 mL/min/1.73m^2^ to ensure the accuracy of the results. Besides, the proportion of female patients was higher in this study. However, we had adjusted for gender in multiple logistic regression analysis to exclude the effect of gender on results (Tables 4 and 5). Therefore, higher female proportion in this study would not affect the accuracy of results.

## 5. Conclusions

In this study, we observed that during 3 years follow-up, the control of body weight, blood glucose, BP, and LDL-C were improved in new-onset T2DM. However, the TG was not well controlled. Further analysis showed that the long-term TG control was very important in delaying the decline of kidney function in the early stage of DKD. It is time to pay more attention to the control of TG in new-onset T2DM.

## Figures and Tables

**Table 1:** Baseline and follow-up characteristics of study participants.

Characteristics	Baseline	Follow-up	*P* value
Female (*n*, %)	195 (68.9%)	195 (68.9%)	1.000
Age (years)	60.68 ± 8.24	63.68 ± 8.24	<0.001
BMI (kg/m^2^)	27.33 ± 3.44	26.30 ± 3.37	<**0.001**
SBP (mmHg)	146.07 ± 19.83	136.30 ± 18.40	<**0.001**
DBP (mmHg)	81.15 ± 10.78	77.98 ± 11.21	<**0.001**
FBG (mmol/L)	6.93 ± 1.82	6.90 ± 1.79	0.823
PBG (mmol/L)	9.72 ± 4.07	11.06 ± 3.88	<**0.001**
HbA_1c_ (%)	7.02 ± 1.22	6.65 ± 1.02	<**0.001**
TG (mmol/L)	1.96 ± 1.35	2.11 ± 1.30	0.187
HDL-C (mmol/L)	1.43 ± 0.29	1.23 ± 0.25	<**0.001**
LDL-C (mmol/L)	3.48 ± 0.96	3.15 ± 0.88	<**0.001**
Creatinine (*μ*mol/L)	64.43 ± 8.65	71.86 ± 11.17	<**0.001**
eGFR (mL/min/1.73 m^2^)	93.90 ± 8.45	85.08 ± 11.26	<**0.001**
Overweight/obesity (*n*, %)	210 (74.2%)	177 (62.5%)	**0.003**
Hypertension (*n*, %)	181 (64.0%)	117 (41.3%)	<**0.001**
Poor glycemic control (*n*, %)	229 (80.9%)	142 (50.2%)	<**0.001**
High TG (*n*, %)	137 (48.4%)	158 (55.8%)	0.077
Low HDL-C (*n*, %)	64 (22.6%)	136 (48.1%)	<**0.001**
Out of target for LDL-C (*n*, %)	243 (85.9%)	208 (73.5%)	<**0.001**

Data are mean ± SD or number (%). BMI: body mass index; SBP: systolic blood pressure; DBP: diastolic blood pressure; FBG: fasting blood glucose; PBG: postprandial blood glucose; TG: triglyceride; HDL-C: high-density lipoprotein cholesterol; LDL-C: low-density lipoprotein cholesterol; eGFR: estimated glomerular filtration rate. Significant *P* values (<0.05) are indicated in bold.

**Table 2:** Characteristics of study participants by △eGFR.

Characteristics		Group 1 (*N* = 94)	Group 2 (*N* = 95)	Group 3 (*N* = 94)
Female (*n*, %)	Baseline	57 (60.6%)	69 (72.6%)	69 (73.4%)

Age (years)	Baseline	59.99 ± 8.85	61.05 ± 7.36	61.00 ± 8.49
	Baseline	27.24 ± 3.31	27.77 ± 3.31	26.98 ± 3.68

BMI (kg/m^2^)	Follow-up	25.70 ± 3.21	26.79 ± 3.40	26.41 ± 3.45
	**△**	-1.35 (-2.40 to -0.46)	**-1.06 (-1.82 to 0.02)** ^**a**^	**-0.57 (-1.74 to 0.31)** ^**a**^
	Baseline	145.00 ± 20.53	148.57 ± 19.51	144.61 ± 19.39

SBP (mmHg)	Follow-up	137.62 ± 20.45	136.22 ± 18.08	135.07 ± 16.57
	**△**	-8.50 (-16.00 to 2.00)	-12.00 (-23.00 to -1.00)	-11.00 (-22.00 to 2.00)
	Baseline	81.76 ± 10.30	81.82 ± 10.61	79.87 ± 11.41

DBP (mmHg)	Follow-up	79.60 ± 11.53	76.62 ± 10.31	77.74 ± 11.67
	**△**	-2.00 (-8.00 to 4.00)	-5.00 (-12.00 to 1.00)	-3.00 (-10.00 to 5.00)
	Baseline	7.13 ± 1.90	6.93 ± 1.77	6.75 ± 1.80

FBG (mmol/L)	Follow-up	6.88 ± 1.56	6.74 ± 1.54	7.08 ± 2.19
	**△**	-0.23 (-0.93 to 0.51)	0.25 (-0.88 to 0.71)	**0.21 (-0.48 to 1.08)** ^**a**^
	Baseline	10.16 ± 4.57	9.77 ± 3.79	9.24 ± 3.78

PBG (mmol/L)	Follow-up	11.28 ± 3.98	10.55 ± 3.26	11.36 ± 4.32
	**△**	1.01 (-1.11 to 3.05)	1.68 (-1.37 to 3.17)	1.77 (-0.22 to 4.35)
	Baseline	7.07 ± 1.40	6.95 ± 1.07	7.03 ± 1.19

HbA_1C_ (%)	Follow-up	6.64 ± 1.10	6.58 ± 0.86	6.74 ± 1.07
	**△**	-0.30 (-0.80 to -0.18)	-0.30 (-0.60 to 0.00)	-0.25 (-0.70 to 0.00)
	Baseline	2.01 ± 1.65	1.85 ± 0.95	2.02 ± 1.38

TG (mmol/L)	Follow-up	1.89 ± 1.22	2.15 ± 1.41	2.28 ± 1.24^**a**^
	**△**	0.01 (-0.42 to 0.34)	**0.18 (-0.23 to 0.55)** ^**a**^	**0.28 (-0.23 to 0.93)** ^**a**^
	Baseline	3.46 ± 0.87	3.57 ± 1.02	3.40 ± 1.00

LDL-C (mmol/L)	Follow-up	3.01 ± 0.78	3.23 ± 0.93	3.21 ± 0.92
	**△**	-0.42 (-0.80 to -0.01)	-0.26 (-0.76 to 0.14)	**-0.19 (-0.77 to 0.41)** ^**a**^
	Baseline	65.12 ± 9.57	63.08 ± 7.48	65.12 ± 8.70

Creatinine (*μ*mol/L)	Follow-up	67.10 ± 9.63	69.62 ± 7.77	78.87 ± 12.11^**ab**^
	**△**	1.80 (-0.10 to 3.80)	**6.40 (4.80 to 7.80)** ^**a**^	**10.50 (7.65 to 16.28)** ^**ab**^
	Baseline	94.65 ± 8.50	94.05 ± 8.40	93.00 ± 8.47

eGFR (mL/min/1.73 m^2^)	Follow-up	92.04 ± 8.56	86.84 ± 8.86^**a**^	76.33 ± 10.14^**ab**^
	**△**	-2.66 (-3.89 to -1.30)	**-7.30 (-8.47 to -5.92)** ^**a**^	**-14.45 (-18.29 to -11.10)** ^**ab**^
	Baseline	69 (73.4%)	71 (74.7%)	70 (74.5%)

Overweight/obesity (*n*, %)	Follow-up	51(54.3%)	63 (66.3%)	63 (67.0%)
	**△**	-18 (19.1%)	**-8 (8.4%)** ^**a**^	**-7 (7.4%)** ^**a**^
	Baseline	59 (62.8%)	64 (67.4%)	58 (61.7%)

Hypertension (*n*, %)	Follow-up	41 (43.6%)	37 (38.9%)	39 (41.5%)
	**△**	-18 (19.1%)	-27 (28.4%)	-19 (20.2%)
	Baseline	71 (75.5%)	79 (83.2%)	79 (84.0%)

Poor glycemic control (*n*, %)	Follow-up	43 (45.7%)	50 (52.6%)	49 (52.1%)
	**△**	-28 (29.8%)	-29 (30.5%)	-30 (31.9%)
	Baseline	41 (43.6%)	45 (47.4%)	51 (54.3%)

High TG (*n*, %)	Follow-up	41 (43.6%)	54 (56.8%)	**63 (67.0%)** ^**a**^
	**△**	0 (0.0%)	**9 (9.5%)** ^**a**^	**12 (12.8%)** ^**a**^
	Baseline	19 (20.2%)	21 (22.1%)	24 (25.5%)

Low HDL-C (*n*, %)	Follow-up	42 (44.7%)	47 (49.5%)	47 (50.0%)
	**△**	23 (24.5%)	26 (27.4%)	23 (24.5%)
	Baseline	85 (90.4%)	79 (83.2%)	79 (84.0%)

Out of target for LDL-C (*n*, %)	Follow-up	67 (71.3%)	68 (71.6%)	73 (77.7%)
	**△**	-18 (19.1%)	-11 (11.6%)	**-6 (6.4%)** ^**a**^

Data are mean ± SD or number (%). BMI: body mass index; SBP: systolic blood pressure; DBP: diastolic blood pressure; FBG: fasting blood glucose; PBG: postprandial blood glucose; TG: triglyceride; LDL-C: low-density lipoprotein cholesterol; eGFR: estimated glomerular filtration rate.

**Table 3:** Spearman correlation analysis of the △ (metabolic indicators) with △eGFR.

Characteristics	*r*	*P*
△BMI	-0.204	**0.001**
△SBP	0.061	0.308
△DBP	0.001	0.986
△TG	-0.155	**0.009**
△HDL-C	-0.112	0.060
△LDL-C	-0.165	**0.005**
△FBG	-0.178	**0.003**
△PBG	-0.100	0.093
△HbA1c	-0.071	0.232

**Table 4:** The associations of baseline and follow-up metabolic abnormalities with the most reduced eGFR.

	Model 1		Model 2	
Variables	OR (95% CI)	*P* value	OR (95% CI)	*P* value
Baseline analysis				
Age (per year)	1.007 (0.977 to 1.038)	0.647	1.007 (0.976 to 1.039)	0.670
Sex (female)	1.380 (0.797 to 2.388)	0.250	1.322 (0.734 to 2.380)	0.353
Overweight/obesity	1.021 (0.579 to 1.799)	0.943	0.969 (0.537 to 1.747)	0.917
Hypertension	0.864 (0.518 to 1.443)	0.577	0.837 (0.494 to 1.418)	0.508
Poor glycemic control	1.369 (0.711 to 2.636)	0.347	1.321 (0.673 to 2.593)	0.419
High TG	1.420 (0.864 to 2.334)	0.166	1.413 (0.833 to 2.398)	0.200
Low HDL-C	1.277 (0.715 to 2.282)	0.409	0.992 (0.520 to 1.893)	0.981
Out of target for LDL-C	0.803 (0.401 to 1.607)	0.535	0.718 (0.351 to 1.469)	0.364
Follow-up analysis				
Age (per year)	1.007 (0.977 to 1.038)	0.647	1.011 (0.979 to 1.043)	0.503
Sex (female)	1.380 (0.797 to 2.388)	0.250	1.335 (0.723 to 2.464)	0.356
Overweight/obesity	1.337 (0.795 to 2.248)	0.273	1.209 (0.697 to 2.097)	0.500
Hypertension	1.009 (0.611 to 1.667)	0.972	0.966 (0.576 to 1.621)	0.897
Poor glycemic control	1.124 (0.685 to 1.844)	0.643	1.023 (0.609 to 1.718)	0.932
High TG	**2.011 (1.200 to 3.369)**	**0.008**	**2.097 (1.199 to 3.670)**	**0.009**
Low HDL-C	1.124 (0.685 to 1.843)	0.645	0.829 (0.464 to 1.482)	0.527
Out of target for LDL-C	1.390 (0.779 to 2.481)	0.264	1.541 (0.835 to 2.845)	0.167

CI: confidence interval. Significant *P* values (<0.05) are indicated in bold. Model 1: all the independent variables were analyzed separately; Model 2: all the independent variables were analyzed simultaneously. Significant *P* values (<0.05) are indicated in bold.

**Table 5:** The association of different TG control groups with the most reduced eGFR.

	Model 1		Model 2		Model 3	
Variables	OR (95% CI)	*P* value	OR (95% CI)	*P* value	0R (95% CI)	*P* value
G1 (*n* = 99)	1		1		1	
G2 (*n* = 26)	1.469 (0.565 to 3.815)	0.430	1.476 (0.565 to 3.855)	0.427	1.552 (0.586 to 4.112)	0.376
G3 (*n* = 47)	2.448 (1.164 to 5.145)	**0.018**	2.432 (1.153 to 5.131)	**0.020**	2.576 (1.198 to 5.539)	**0.015**
G4 (*n* = 111)	2.090 (1.144 to 3.818)	**0.017**	2.028 (1.106 to 3.718)	**0.022**	2.184 (1.133 to 4.207)	**0.020**

OR: odds ratio; CI: confidence interval. Significant *P* values (<0.05) are indicated in bold. Model 1: not adjusted. Model 2: adjusted for age and gender. Model 3: adjusted for age, gender, overweight/obesity, hypertension, poor glycemic control, high TG, low HDL-C, and out of target for LDL-C.

## Data Availability

The data used to support the findings of this study are available from the corresponding author upon request.
